# An assessment of the magnitude, parallelism, and asymmetry of micro-implant-assisted rapid maxillary expansion in non-growing patients

**DOI:** 10.1186/s40510-020-00342-4

**Published:** 2020-11-23

**Authors:** Islam Elkenawy, Layla Fijany, Ozge Colak, Ney Alberto Paredes, Ausama Gargoum, Sara Abedini, Daniele Cantarella, Ramon Dominguez-Mompell, Luca Sfogliano, Won Moon

**Affiliations:** Division of Growth and Development, Section of Orthodontics, School of Dentistry, Center for Health Science, University of California at Los Angeles, Room 63-082 CHS, 10833 Le Conte Ave, Box 951668, Los Angeles, CA 90095-1668 USA

## Abstract

**Background and objectives:**

Micro-implant-assisted expanders have shown significant effects on the mid-face, including a degree of asymmetry. The aim of this study is to quantify the magnitude, parallelism, and asymmetry of this type of expansion in non-growing patients.

**Methods:**

A retrospective study on a sample of 31 non-growing patients with an average age of 20.4 years old, with cone beam computed tomography images taken before and right after expansion using maxillary skeletal expander (MSE) were assessed for skeletal expansion at three landmarks bilaterally.

**Results:**

Average magnitude of total expansion was 4.98 mm at the anterior nasal spine (ANS) and 4.77 mm at the posterior nasal spine (PNS) which showed statistical significance using a paired *t* test with *p* < 0.01. Average expansion at the PNS was 95% of that at the ANS. The sample was divided into symmetric (*n* = 15) and asymmetric (*n* = 16) based on the difference in expansion at the ANS, with 16 out of 31 patients exhibiting statistically significant asymmetry.

**Conclusions:**

MSE achieves distinctly parallel expansion in the sagittal plane but can exhibit asymmetrical expansion in the transverse plane.

## Highlights

- MSE-type expander can expand non-growing patients with an average of 5 mm at ANS
- Mean expansion at PNS was 4.7 mm, giving 96% parallelism in the sagittal direction
- 50% of the sample size exhibited asymmetric expansion in the transverse plane
- Within the asymmetric samples, the split was on average 2.22 mm more on one side
- Possible correlation between the direction of asymmetry and unilateral crossbites

## Introduction

Transverse maxillary deficiency (TMD) is a common malocclusion that is diagnosed when the maxilla is narrow in relation to the mandible [1]. Patients with TMD often present with unilateral or bilateral posterior crossbite, anterior crowding, and large buccal corridors upon smiling [2]. Adequate transverse maxillary dimension is essential for stable, well-balanced, and proper functional occlusion. Traditionally, rapid palatal expander (RPE) is often considered the appliance of choice to treat patients diagnosed with TMD to increase transverse maxillary dimension. It is usually performed in childhood or adolescence before the midpalatal suture has matured [3, 4]. With age, the midpalatal suture becomes more interdigitated and denser and is believed to be fully fused by 15–19 years old [5]. Once the midpalatal suture matured, RPE appliances become less effective in achieving basal skeletal expansion and the force they apply may lead to dentoalveolar tipping [6].

**Fig. 1:**
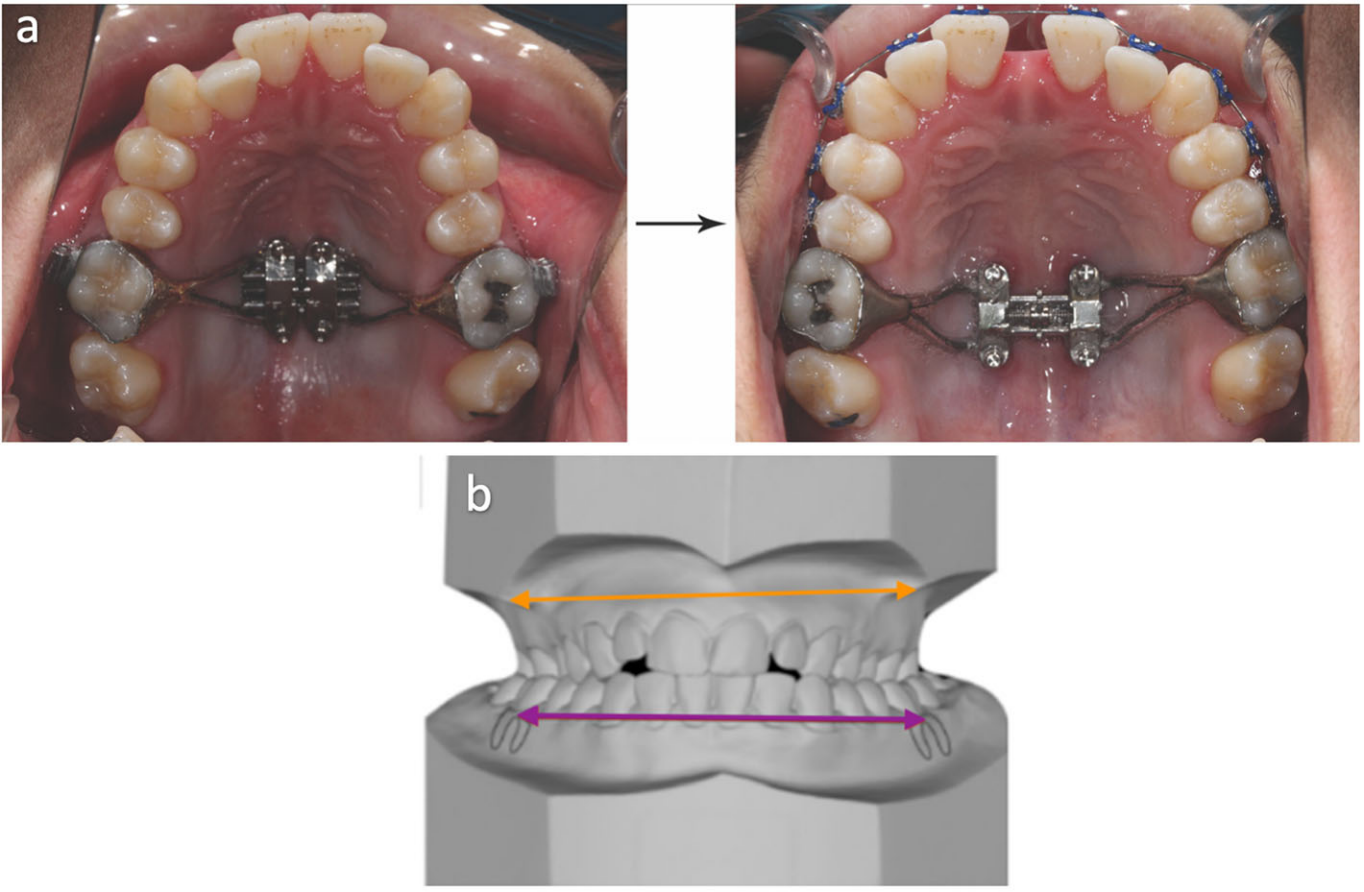
**a** Occlusal view before and after expansion using MSE, with a clinically visible anterior diastema. **b** Showing the method of diagnosing transverse maxillary skeletal deficiency

Recently, bone-borne expanders utilizing temporary anchorage devices (TADs) such as maxillary skeletal expander (MSE) (Fig. 1) are being used to reduce the drawbacks of dentoalveolar tipping caused by RPE [7]. Although MSE has shown to lead to more parallel expansion in the anterior-posterior dimension [8, 9], only few studies have quantified the amount of expansion, including the degree of parallelism, and none have focused on non-growing patients exclusively.

Current literature studying the skeletal effects induced by traditional RPE has not addressed the variation in the symmetry of expansion in the transverse dimension. However, with the increased use of MSE to successfully expand adults, whose sutures are believed to be fused [10], clinically significant asymmetry of expansion has also been documented [8]. In addition, surgically assisted rapid palatal expansion (SARPE) was also shown to exhibit significant asymmetry in the transverse direction as recently documented [11].

These new discoveries have raised a number of questions and hypotheses about different factors that could ascribe to this asymmetric expansion, in particular, the difference in bone density of the circum-maxillary sutures and the surrounding structures, presence or absence of crossbite, and difference in the morphology of bones on each side. Although, in one study, suture density ratio has been proposed as a possible predictor for the amount of orthopedic expansion [12], the nature of this asymmetry is yet to be adequately studied and documented, as its clinical significance remains poorly elucidated.

The use of cone beam computed tomography (CBCT) provides clinicians with exceptional detailed images, that have not been explored in previous studies which utilized traditional two-dimensional imaging [3]. The use of 3D multi-planar software allows for more robust analysis of the nature of the pattern of expansion, in addition to helping clinicians make an accurate diagnosis [13, 14]. Thus, as clinicians, it is important to assess asymmetry of expansion to be able to devise treatment plans that will create an achievable balance for the patient.

For that reason, the present study was undertaken to quantify the magnitude, parallelism, and asymmetry of micro-implant-assisted rapid maxillary expansion in non-growing patients.

## Materials and methods

### Study design

Institutional review board approval (IRB number 17-000567) was granted by the University of California, Los Angeles (UCLA), to perform this retrospective study. The study included 31 non-growing subjects (cervical vertebral maturation stage V) who have undergone expansion using MSE (BioMaterials Korea, Inc.) with a mean age of 20.4 ± 3.2 years (range 17–27 years). TMD was diagnosed by measuring the basal bone widths discrepancy based on Andrews’ analysis of six elements (Andrews) [15]. Thirteen patients had bilateral posterior crossbite, eleven patients had unilateral crossbite, and seven patients had maxillary transverse deficiency without posterior dental crossbite. All patients were treated at the same institution. MSE treatment was initiated and completed prior to bonding of brackets or other orthodontic appliances, and CBCT images were obtained before, and right after expansion was complete.

### Inclusion criteria

The inclusion criteria used in this retrospective study include patients who were diagnosed with maxillary transverse deficiency [15] with cervical vertebral maturation stage (CVMS) V and had no previous orthodontic treatment [8].

Maxillary width was determined based on the distance between the right and left most concave point on buccal side, corresponding to the first molars’ mesio-buccal cusp level. Mandibular width was determined based on the distance between the right and left mandibular WALA ridge, corresponding to the first molars’ mesio-buccal groove level. WALA ridge, based on Andrews’ analysis of six elements, was formed by the most prominent part of the alveolar process on buccal side [15]. Diagnosis of transverse maxillary deficiency was conducted by calculating the difference between maxillary and mandibular width, as shown in Fig. 1b.

Exclusion criteria involved patients with any systemic diseases as well as craniofacial syndromes that could change the outcome of the treatment. The patients who met the abovementioned criteria composed our sample size.

### Expander design and activation rates

The same MSE design (Fig. 1a) was used for all patients. The MSE contains a central jackscrew unit, positioned at the posterior palate with 4 microimplants size 1.5 × 11 mm, and attaches to the molars with connecting arms and molar bands. The activation protocol was set at two activations (0.40 mm) per day until a diastema appears at which then the rate was switched to one activation per day. Expansion was complete when the maxillary basal bone width was greater than the mandibular width. Average duration of expansion was 35 ± 10 days. After proper maxillary expansion was achieved, the MSE stayed affix for another 6 months for bone formation.

### 3D analysis

CBCT scans were taken prior to expansion and within 3 weeks following completion of maxillary expansion on all patients. The post-expansion scans were always taken prior to bonding of brackets or any orthopedic appliance. These two factors ensured that the sutures remained patent, prior to bone formation, when CBCTs were taken, which allows for accurate measurements.

All CBCT scans were taken by a NewTom 5G scanner in an 18 × 16 field of view with a 14-bit gray scale and with a voxel size of 0.3 mm. Scan times were 18 s (3.6 s emission time), with 110 kV, and utilized an automatic exposure control that adjusted the milliampere based upon the patient’s anatomic density. Five hundred thirty-eight axial slices with 609 × 609 resolution and a slice thickness and increment of 0.3 mm and pixel spacing of 0.3 mm were obtained. The fusion module by OnDemand 3D (Cybermed Inc., Korea) was used to superimpose the post-expansion CBCT on the pre-expansion CBCT using the anterior cranial base as a stable reference on non-growing patients, as proposed by Cevidanes et al. [16]. This is a fully automated tool by the software that relies on grayscale values and multiple iterations of best fit, to circumvent errors related to the operator. This method was verified for accuracy by Weissheimer et al. [12, 16].

**Fig. 2:**
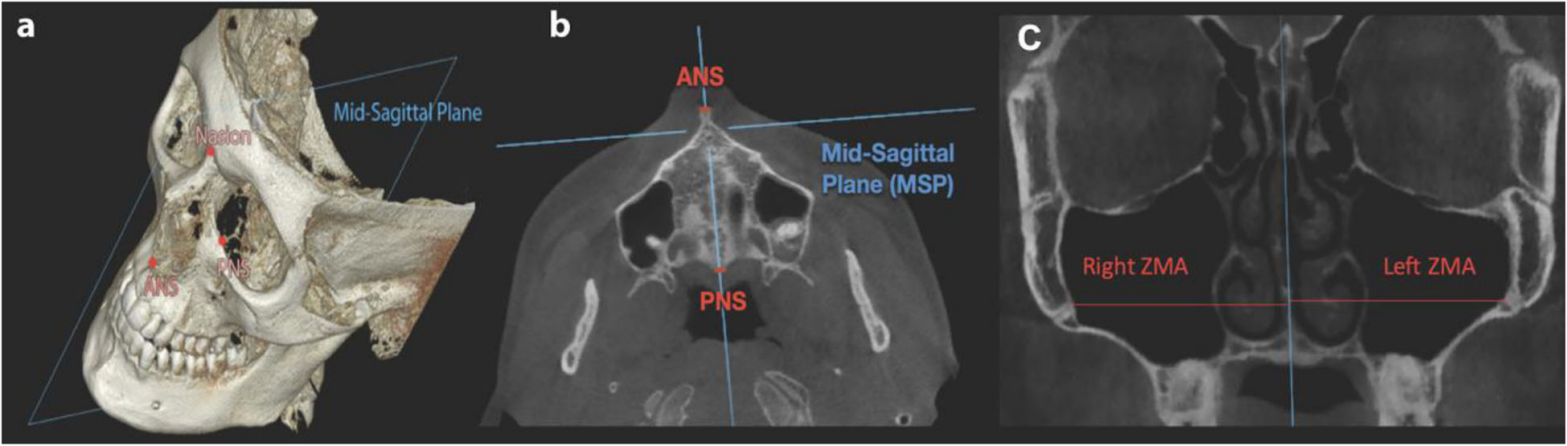
**a** CBCT image showing mid-sagittal plane (MSP) on subject’s initial CBCT using OnDemand (Cybermed, Korea). ANS, PNS, and nasion can be viewed as separate skeletal landmarks on the MSP. **b** Axial slice at pre-expansion with vertical line passing through ANS and PNS. **c** Coronal view of pre-expansion CBCT displaying measurements from the MSP to both the right and left ZMA. Right and left ZMA landmarks indicated in red at the most medial-superior location of the zygomatic-maxillary suture

**Fig. 3:**
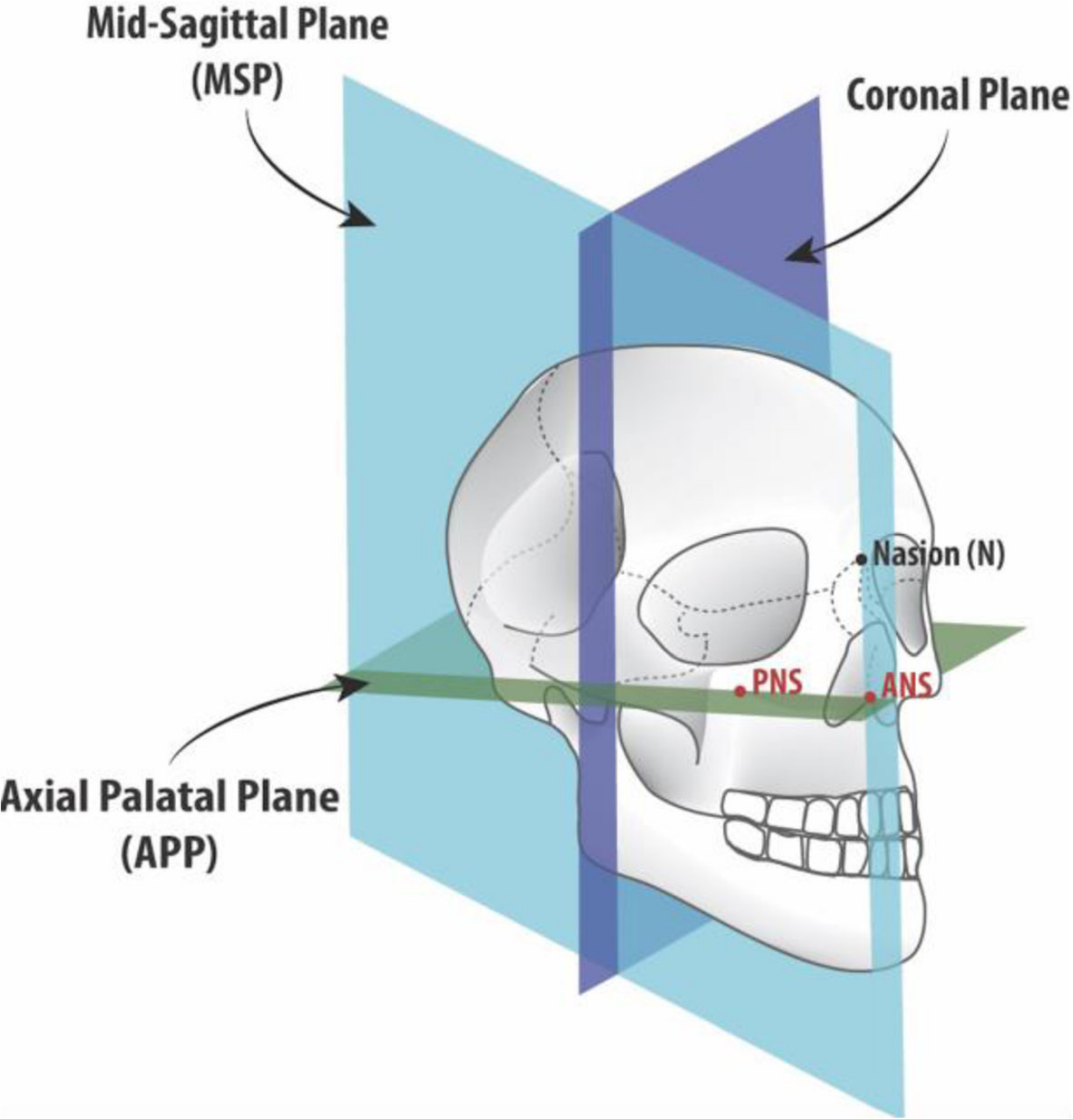
Illustration of the two main reference planes: mid-sagittal plane (MSP) and axial palatal plane (APP) and coronal plane added for orientation. Planes are identified in the pre-expansions CBCT and become the reference lines to measure the displacement of skeletal landmarks in the post-expansion CBCT. Note that the MSP passes through the ANS, PNS, and nasion on the pre-expansion CBCT. The APP also passes through the ANS and PNS and is perpendicular to the MSP, in the pre-expansion CBCT

The mid-sagittal plane (MSP) was utilized as the plane of reference for all measurements made as it was found to be the most accurate plane to quantify lateral maxillary expansion based on a recent publication [17]. MSP is a plane passing through the nasion (N), anterior nasal spine (ANS), and posterior nasal spine (PNS), generated on the pre-expansion CBCT (Figs. 2 and 3). It is created based on the pre-expansion CBCT image and remains a fixed reference to measure post-expansion changes with the pre- and post-expansion CBCT scans superimposed. The axial palatal plane (APP) is perpendicular to the MSP and passes through the ANS and PNS (Figs. 2 and 3). Lateral measurements were made on axial sections that were created at the level of this plane.

### Measurements at the mid-sagittal plane

**Fig. 4:**
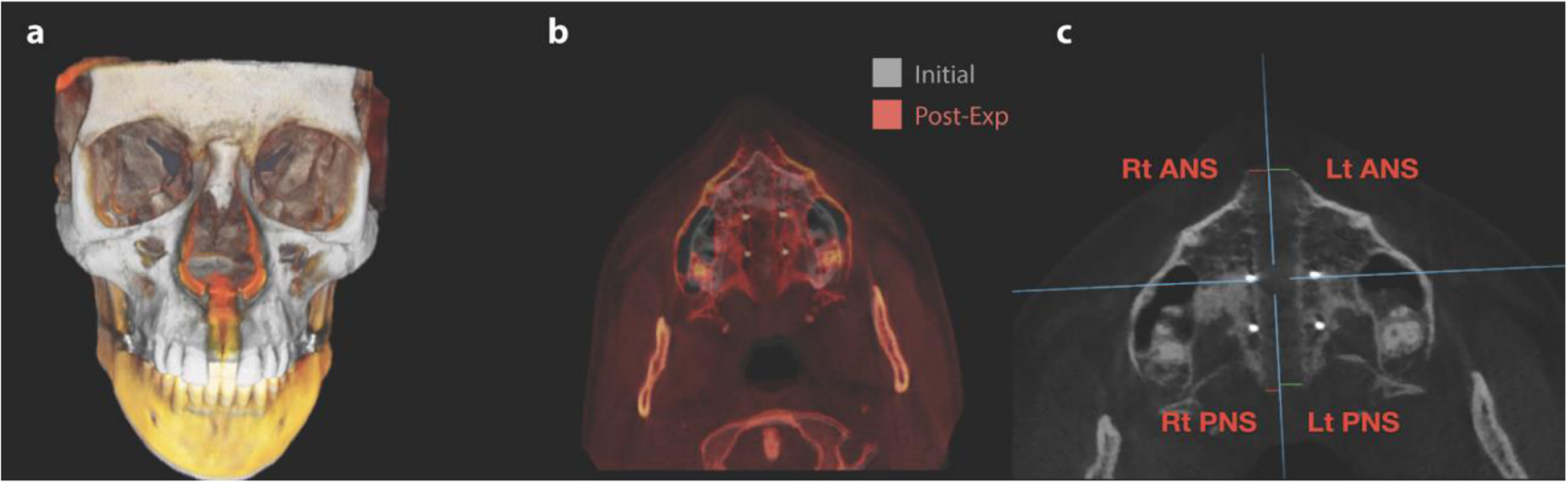
**a** 3D superimposition of pre- and post-expansion on an individual patient demonstrating changes to mid-face. **b** Axial view of superimposed pre- and post-expansion. **c** Axial view of post-expansion CBCT showing measurement from MSP to both the right and left ANS and PNS landmarks with apparent asymmetry

To accurately quantify the extent of skeletal expansion, the method proposed by Cantarella et al. [8] was used with slight modifications. In the present study, the MSP has been used to study the split of the midpalatal suture. Lateral displacement was measured from the right or left sides to MSP for skeletal landmarks, including anterior nasal spine (ANS), posterior nasal spine (PNS), and zygomaticomaxillary point (ZMA). The MSE splits the midpalatal suture, in which the previously singular ANS and PNS skeletal landmarks will be divided into their respective right (Rt) and left (Lt) landmarks in each patient following skeletal expansion (Fig. 4). In the pre-expansion CBCT, the MSP passes through the ANS and PNS, and in the post-expansion CBCT, linear measurements were made from the ANS and PNS skeletal landmarks on right and left sides to the MSP in the axial cuts at the level of the APP. The post-expansion Rt and Lt distances of each landmark represent the lateral expansion of each side. Zygomaticomaxillary point (ZMA) represents the most medial aspect of the zygomaticomaxillary suture when viewed in the coronal zygomatic section (Fig. 2c). This section passes through the lowest point of the zygomaticomaxillary sutures and the uppermost point of the frontozygomatic sutures also known as the coronal zygomatic section [18]. Similarly, in pre- and post-expansion CBCT images, the distance from the right and left zygomaticomaxillary suture was measured to the mid-sagittal plane at the level of the coronal plane. The sum of displacements of right and left sides represented the total amount of expansion.

### Magnitude of expansion and deviation

For all patients, total expansion was measured by adding the amount of expansion on both sides of the MSP (Rt + Lt Post-expansion − Rt + Lt Pre-expansion) for the ANS, PNS, and ZMA. For all three skeletal landmarks, if the right and left halves did not expand equally, then a deviation was present. Furthermore, in order to quantify the amount of potential deviation, the data was re-organized to calculate the absolute difference between the greater and lesser side denoted as “Deviation” (without assigning them with a positive and negative sign) for all measurements; for example, ANS Deviation = [Greater ANS − Lesser ANS].

Subsequently, the difference between right and left was calculated for each and denoted as “Asymmetry”. For instance, ANS Asymmetry = [Right ANS − Left ANS]. If the result of this calculation was positive then it indicates that the right side was larger than the left side after expansion in ANS and vice versa. This was done in order to quantify the direction of the asymmetry.

The movement at the ANS was chosen as a reference to further group the subjects because changes at the ANS have a smaller standard deviation for total expansion. Additionally, another publication chose the movement of the ANS as a parameter to analyze expansion because changes at ANS reflect modifications in the anterior part of the maxilla more closely. Therefore, it can have a larger impact on the soft tissues of the face [8, 19].

### Post-expansion grouping of patients

To further understand transverse asymmetry post-expansion, the absolute value of the difference between the right and left sides was calculated. This was done by giving three positive values for ANS, PNS, and ZMA each. Based on the standard deviation (SD) of the ANS deviation from the 31 patients, the subjects were split into 2 groups, symmetric and asymmetric. Further analysis was conducted on each group.

### Statistical analysis

Sample size power analysis was calculated with 80% power at an effect size of 0.5, and an alpha value of 0.05 using G*power 3.1.9.3 software (Franz Faul, Universität Kiel, Germany) [8, 20]. Descriptive statistics, including means and standard deviations, were calculated for the data. With the 31 patients, a paired *t* test was performed to compare the pre- and post-expansion measurements at the ANS, PNS, and ZMA based on central limit theorem. Pearson correlation coefficient (*R*) was calculated for the correlation among total ANS and PNS expansion in all patients. Within the asymmetric group of patients, the Mann-Whitney *U* test (alpha = 0.05) was utilized to compare the greater vs lesser measurements at the ANS, PNS, and ZMA. To evaluate interobserver reliability, all measurements in 10 patients were repeated by a different investigator. A paired *t* test was performed, and limits of agreement and an intra-class correlation coefficient (ICC; one-way random model, absolute agreement) were calculated. Statistical analyses were performed in SPSS Statistics 25 (IBM Corporation, Armonk, NY, USA). A 95% confidence level (*p* < 0.05) was considered statistically significant.

## Results

### Total expansion/magnitude of expansion

Initial values of ANS and PNS before MSE were 0 mm, as they are a singular landmark prior to expansion (Table 1). MSE expansion resulted in significant increase in transverse dimension at the ANS, PNS, and ZMA with *p* values < 0.001 (Tables 1 and 2). For magnitude of expansion, the greatest average displacement was seen at the ANS, 4.98 mm, compared to the PNS and ZMA (Tables 1 and 2). The largest range of expansion was seen at the PNS (0–13.3 mm), taking into consideration that two patients did not display any measurable expansion at the PNS. This could be a result of either lack of actual split or due to bone density thresholds not permitting proper visualization of the split. The difference in the post-expansion lateral measurements at the ANS, PNS, and ZMA were significantly greater than their respective pre-expansion values (*p* < 0.001). Following MSE treatment in all patients, the average expansion at the ANS was 4.98 mm, with the PNS displaying 4.77 mm of average expansion. The smallest change in average expansion was observed at the ZMA, with a mean magnitude of 3.99 mm (Table 2, Figs. 4 and 6).

**Table 1:** Total ANS and PNS was calculated as right plus left in millimeter

	Initial		Post-expansion		Treatment change		
	Mean	SD	Mean	SD	Mean	SD	*p* value
Total ANS expansion	0	0	4.98	1.94	4.98	1.94	< .0001
Total PNS Expansion	0	0	4.77	2.65	4.77	2.65	< .0001

**Table 2:** Showing total expansion in ZMA (millimeters)

	Pre-expansion			Post-expansion			Total expansion, RT+LT ZMA		Treatment change	*p* value
	RT	LT	Diff	RT	LT	Diff	Pre	Post		
Mean	40.36	40.16	0.20	42.46	42.05	0.41	80.53	84.51	3.99	< .0001
SD	-	-	1.55	-	-	2.33	4.91	5.34	1.60	

*RT* right, *LT* left

### Parallelism

To evaluate the parallelism in the sagittal dimension, the ratio of the amount of expansion at the ANS to the amount of expansion at PNS was measured. PNS expansion was 4.77 mm and ANS expansion was 4.98 mm giving a 95.7% parallel expansion in the anterior-posterior dimension. Correlation coefficient between total ANS expansion and total PNS expansion was *R*^2^ = 0.69.

### Deviation

**Fig. 5:**
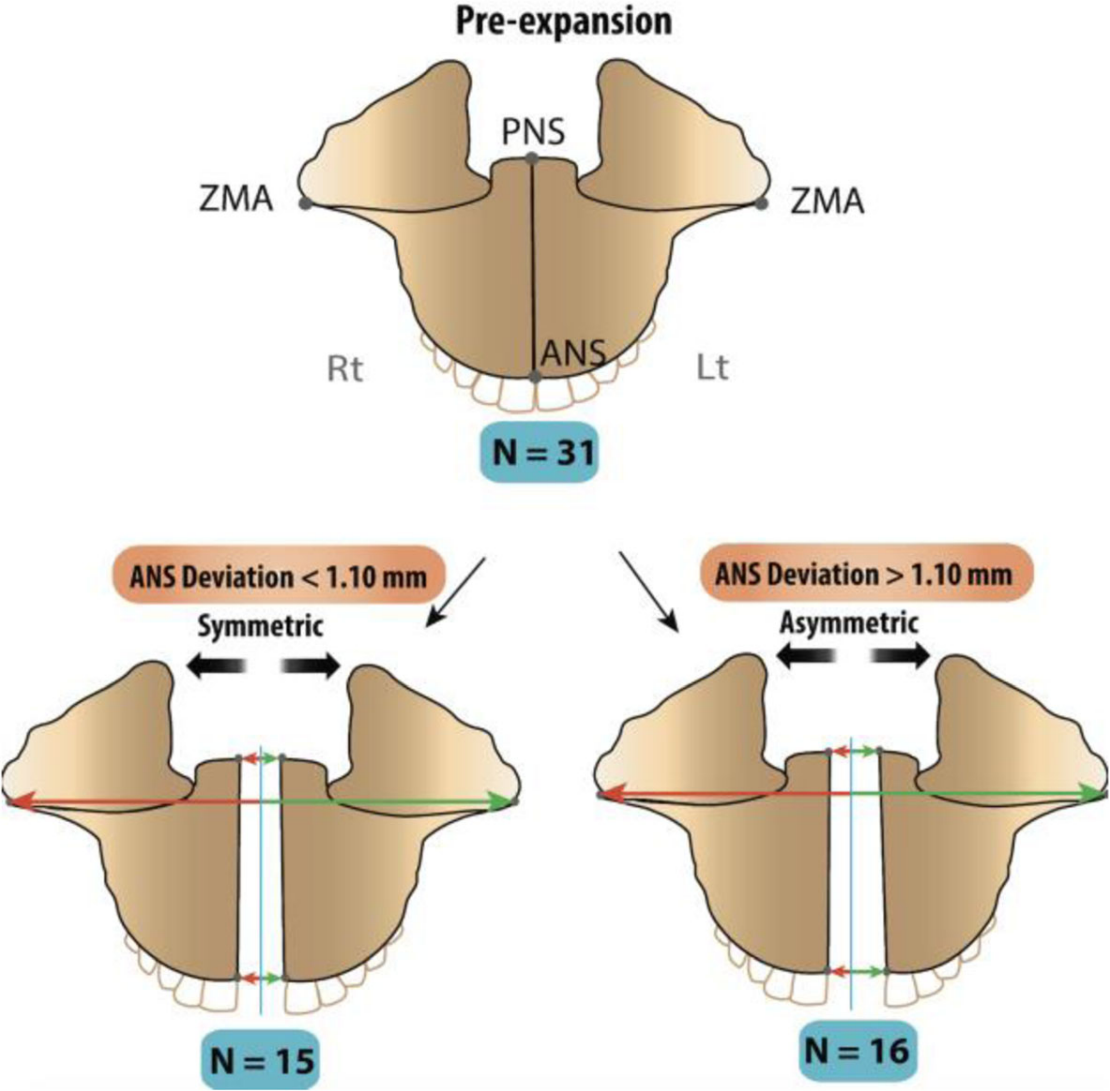
Illustration of post-expansion symmetric and asymmetric groups after MSE treatment. Bottom left: Example of almost parallel transverse expansion, where the ANS deviation was less than 1.1 mm. Bottom right: Example of transverse asymmetry where lateral movement of left maxilla is greater than that of the right, where the ANS deviation was more than 1.1 mm. Blue line represents the MSP. Within each group, the direction of expansion (right or left) was not considered, and instead all expansion measurements were sorted into either “Greater” or “Lesser” values for the purpose of evaluating the amount of absolute deviation

The prior singular ANS and PNS skeletal landmarks can now be visualized as having a right (Rt) and left (Lt) in each subject (Figs. 4c and 5). The absolute value of the difference in expansion between the two sides was calculated; this value represents the deviation for the ANS or PNS. The SD of the ANS deviation (Rt ANS − Lt ANS absolute) was 1.1 mm as seen in Table 3. The subjects were then divided into 2 groups based on their ANS deviation values. From 31 total subjects, 15 had an ANS deviation less than 1.10 mm, which were placed in the symmetric group. The other 16 subjects had an ANS deviation greater than 1.10 mm and were placed in the asymmetric group (Fig. 5). To validate the grouping, a *t* test was calculated comparing the greater versus lesser ANS expansion across both symmetric and asymmetric groups. Within the symmetric group, the difference between the greater and lesser values was not statistically significant (*p* > 0.05) (Table 5).

**Table 3:** ANS, PNS, and ZMA deviation values for all patients

	Mean (mm)	SD (mm)	*p* value
ANS deviation |Rt-Lt|	1.37	1.10	0.414
PNS deviation |Rt-Lt|	1.16	0.99	0.421
ZMA deviation |Rt-Lt|	.413	2.34	0.421

**Fig. 6:**
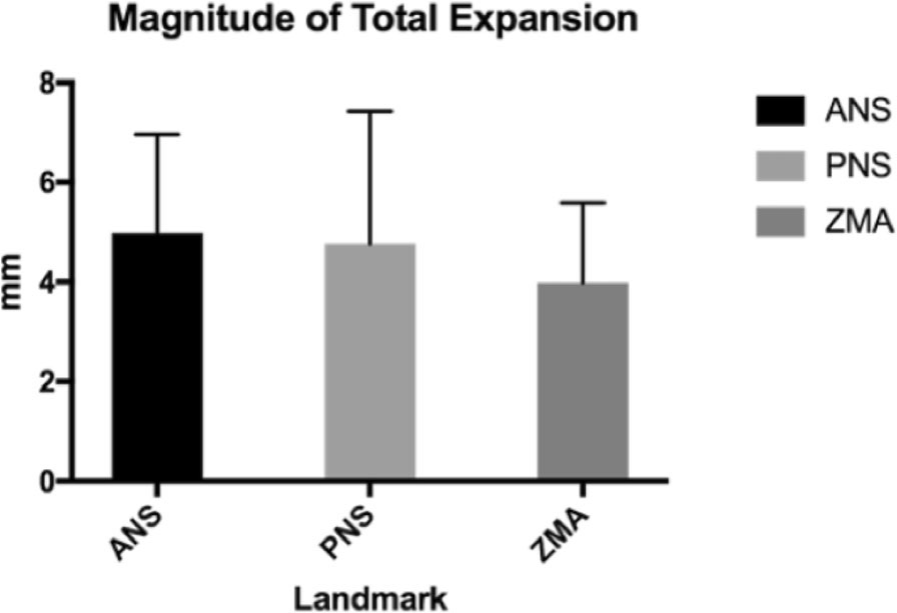
Plot comparing mean magnitude of expansion at the three main landmarks: ANS, PNS, and ZMA

In the asymmetric group, there was a statistical significance between the greater and lesser ANS measurements verifying accurate grouping with *p* < .0001 (Table 4). Based on this sample size, the percentage of patients exhibiting statistically significant ANS deviation representing asymmetry at a magnitude of at least 1.1 mm was 51% (Table 5, Fig. 6).

**Table 4:** Asymmetric group mean for greater and lesser expansion (millimeters) at ANS, PNS, and ZMA

	Treatment change		
	Mean	SD	*p* value
Greater ANS	4.08	1.22	< .0001
Lesser ANS	1.86	0.97	
ANS deviation	2.22	0.89	
Greater PNS	3.81	1.44	< .0001
Lesser PNS	2.04	1.55	
PNS deviation	1.77	1.11	
Greater ▲ZMA	2.83	1.23	< .05
Lesser ▲ZMA	1.53	0.85	
▲**ZMA deviation**	1.3	1.18

**Table 5:** Symmetric group mean for greater and lesser expansion (millimeters) at ANS, PNS, and ZMA

	Treatment change		
	Mean	SD	*p* value
Greater ANS	1.98	0.86	> .05
Lesser ANS	1.97	0.58	
ANS deviation	0.46	0.29	
Greater PNS	1.93	1.14	> .05
Lesser PNS	1.73	1.15	
PNS deviation	0.58	0.47	
Greater ZMA	2.09	0.96	> .05
Lesser ZMA	1.42	0.88	
▲**ZMA deviation**	1.08	0.67

When using a non-parametric paired *t* test, within the asymmetric group (*N* = 16), all three landmarks (ANS, PNS, and ZMA) showed statistically significant difference when comparing greater to lesser values for each patient. The greatest deviation from greater to lesser was observed at ANS with a mean of 2.22 mm, followed by PNS (1.77 mm), and the least was at ZMA expansion (1.3 mm) (Table 4).

In asymmetric group, there was an overall left side dominant expansion in ANS. Ten out of 16 patients showed left side dominance and 6 out of 16 patients demonstrated right side dominance after expansion (Table 6). The average expansion with the right side and the left side dominant patients was 2.21 mm and − 2.21 mm, respectively (Table 7).

**Table 6:** Asymmetric group direction of expansion based on ANS asymmetry

Right side dominance	Left side dominance	Total number of patients in asymmetric group	*p* value
6/16 (37.5%)	10/16 (62.5%)	16	1.000

**Table 7:** Mean values for right and left side asymmetry in ANS (millimeters) for the asymmetric group

Parameter	Mean	SD	*p* value
Right side dominant expansion in ANS	2.21	0.93	0.845
Left side dominant expansion in ANS	-2.21	0.92	0.687

For the considered parameters, the ICC value was 0.97 or higher indicating that measurements were very reliable (Table 8).

**Table 8:** Intra-class correlation coefficients (ICC) of the parameters

Parameter	ICC
Total ANS expansion	0.980
Total PNS expansion	0.976
Total ZMA expansion	0.979
ANS deviation for all patients	0.984
PNS deviation for all patients	0.982
ZMA deviation for all patients	0.974
ANS deviation for asymmetric group	0.975
PNS deviation for asymmetric group	0.981
ZMA deviation for asymmetric group	0.980
ANS deviation for symmetric group	0.978
PNS deviation for symmetric group	0.979
ZMA deviation for symmetric group	0.973

## Discussion

Although previous literatures have evaluated the success of TAD-assisted expanders and their magnitudes, little is known about their effects on non-growing patients where the midpalatal suture was believed to be fused.

The results in this study support previous findings described by Cantarella et al. [8] in the pilot study conducted on 15 patients who were all of post-pubertal age (greater than CS4). Despite the increased number of samples, and the more limited inclusion criteria to only CVMS V, the difference in total expansion exhibited in this study compared to Cantarella’s is minimal. Across the 31 non-growing patients, the average amount of expansion was 4.98 mm at ANS and 4.77 mm at PNS, while the previous pilot studies showed 4.8 mm at ANS and 4.3 mm at PNS [8]. This validation of results in non-growing patients shows a high success rate of adequate expansion in adults. A third landmark, ZMA, was added to be able to measure the amount of expansion from the coronal perspective in addition to the axial slices, and it represents a more lateral and more superior anatomic point illustrating zygomatic expansion.

To analyze the sagittal parallelism, the ratio between expansion at PNS in relation to expansion at ANS was found to be 95.7% indicating that the expansion achieved was highly parallel in the sagittal plane, despite the previously proposed fusion of the pterygopalatine sutures. As previously observed in two separate studies, transverse asymmetry following MSE expansion was present both in the skeletal structure, as well as in the soft tissue of the face [8, 19].

When analyzing transverse asymmetries due to expansion, the wide variation with both the symmetric and asymmetric patients makes the averaged value less meaningful. Therefore, subgrouping was performed using the standard deviation of ANS deviation as a threshold for defining symmetric and asymmetric expansion. Another potential bias is that, within the asymmetric group, some expand to the right while some to the left, and the magnitude of asymmetry can be underestimated when the averaging of all discrepancies is used because the right dominant expansion will cancel the impact of left dominant expansion. When calculating ANS deviation, the absolute value was used to negate any bias resulting from whether each suture expanded more to the right or to the left.

When analyzing the entire sample of 31 patients, ANS mean deviation was 1.37 mm, while PNS deviation was 1.16 mm. This measurement indicates the amount by which one side of the ANS expanded more than the other, and these results support previous studies (Table 3).

However, after the subgrouping was done as shown in Fig. 5, only 15 patients were deemed symmetric based on having an ANS deviation of less than 1.1 mm (SD), while the remaining 16 were considered asymmetric, giving a percentage of asymmetry at about 50%. Within each group, the right and left denominations were erased and replaced by “Greater” and “Lesser” giving two values for each landmark, irrelevant of its location. *t* tests were used to validate the subgrouping where in the symmetric group. There was no statistical significance between greater and lesser values for ANS, PNS, or ZMA. While in the asymmetric group, *p* value was less than 0.01. Within the asymmetric group of 16 patients, the values of asymmetry increased significantly when compared to total size comparisons. The amount of asymmetry was highest at ANS, followed by PNS then ZMA. ZMA having the least amount of asymmetry could be attributed to it being closer to the center of rotation of expansion; this also validates the rotational theory regarding expansion as previously studied [18].

The magnitude of asymmetry at ANS of 2.22 mm is almost double of what was previously discovered when assessing the sample as a whole, including symmetrically expanded patients. While in this study, among the total population (*n* = 31), the average magnitude of asymmetry was 1.37 mm, and the range of ANS deviation from one side to the other widely extended from 0.04 to 4.4 mm. Accordingly, clinical significance can vary greatly depending on the actual magnitude of asymmetrical expansion. While asymmetry of expansion was not assessed frequently in the past, another study looking at asymmetry in patients undergoing surgically assisted bone-borne expansion, or SARME, showed asymmetry of more than 3 mm in at least 55% of the patients [11]. This indicates a similar frequency of asymmetry, but a greater magnitude than what is exhibited using MSE. This difference in magnitude could be attributed to the larger magnitude of total expansion usually performed using the surgical technique; a study comparing percentages could be conducted for a more accurate comparison, rather than linear measurements.

In our study, the asymmetrically expanded group showed left side tendency in transverse plane. However, it is not yet known why some patients exhibit asymmetry, and whether it can be controlled or not. Numerous factors can be considered such as the differential bone density at the sutures and their surrounding bones, the stability of TADs, initial asymmetry of the craniofacial skeleton, and the pattern of crossbite. These possible factors can bring clinicians closer to finding an answer by eliminating some of the variables, as well as aiding future research.

The limitations of this study include the need for subgrouping which reduces the sample size. Although the size remains within the required number based on power analysis, the clinical impact is weakened due to the subgrouping necessary to perform accurate analysis regarding the asymmetry of expansion. Future research is required to search for other possible factors that could explain why some patients exhibit transverse asymmetry with expansion while others do not.

## Conclusions

Although MSE is a recommended alternative for expansion in mature patients, expansion was not always symmetric in the transverse plane, with 16 out of 31 of the patients achieving statistically significant asymmetric expansion.

Expansion achieved using MSE is 96% parallel in the sagittal direction.

Among the asymmetric patients, on average, one half of ANS moved more than the contralateral one by 2.22 mm.

## Data Availability

Data of the present study will not be shared because the same data and materials will be used in further publications where the analysis of different mid-face bones and sutures will be presented.

## Abbreviations

TMD: Transverse maxillary deficiency
CBCT: Cone beam computed tomography
MSE: Mid-facial skeletal expander
RPE: Rapid palatal expansion
SARPE: Surgically assisted rapid palatal expansion
IRB: Institutional Review Board
CVMS: Cervical vertebral maturation stage
MSP: Mid-sagittal plane
ZMA: Zygomaticomaxillary point
ANS: Anterior nasal spine
PNS: Posterior nasal spine
TAD: Temporary anchorage device
APP: Axial palatal plane

## References

[1] Betts NJ, Vanarsdall RL, Barber HD, Higgins-Barber K, Fonseca RJ (1995). Diagnosis and treatment of transverse maxillary deficiency. Int J Adult Orthodon Orthognath Surg.

[2] Ramires T, Maia RA, Barone JR (2008). Nasal cavity changes and the respiratory standard after maxillary expansion. Br J Otorhinolaryngol.

[3] Haas AJ (1965). The treatment of maxillary deficiency by opening the midpalatal suture. Angle Orthod.

[4] Wertz R, Dreskin M (1977). Midpalatal suture opening: a normative study. Am J Orthod.

[5] Persson M, Thilander B (1977). Palatal suture closure in man from 15 to 35 years of age. Am J Orthod.

[6] Gurel HG, Memili B, Erkan M, Sukurica Y (2010). Long-term effects of rapid maxillary expansion followed by fixed appliances. Angle Orthod.

[7] Paredes N, Colak O, Sfogliano L (2020). Differential assessment of skeletal, alveolar, and dental components induced by microimplant-supported midfacial skeletal expander (MSE), utilizing novel angular measurements from the fulcrum. Prog Orthod.

[8] Cantarella D, Dominguez-Mompell R, Mallya SM (2017). Changes in the midpalatal and pterygopalatine sutures induced by micro-implant-supported skeletal expander, analyzed with a novel 3D method based on CBCT imaging. Prog Orthod.

[9] Colak O, Paredes NA, Elkenawy I (2020). Tomographic assessment of palatal suture opening pattern and pterygopalatine suture disarticulation in the axial plane after midfacial skeletal expansion. Prog Orthod.

[10] Spillane LM, McNamara JA (1995). Maxillary adaptation to expansion in the mixed dentition. Semin Orthod.

[11] Huizinga MP, Meulstee JW, Dijkstra PU, Schepers RH, Jansma J (2018). Bone-borne surgically assisted rapid maxillary expansion: a retrospective three-dimensional evaluation of the asymmetry in expansion. J Craniomaxillofac Surg.

[12] Weissheimer A, Menezes LM, Koerich L, Pham J, Cevidanes LH (2015). Fast three-dimensional superimposition of cone beam computed tomography for orthopaedics and orthognathic surgery evaluation. Int J Oral Maxillofac Surg.

[13] Anison JJ, Rajasekar L, Ragavendra B (2015). Understanding asymmetry - a review. Biomed Pharmacol J.

[14] Netherway DJ, Abbott AH, Gulamhuseinwala N (2006). Three-dimensional computed tomography cephalometry of plagiocephaly: asymmetry and shape analysis. Cleft Palate Craniofac J.

[15] Andrews LFAW (2000). The six elements of orofacial harmony. Andrews J Orthod Orofac Harmony.

[16] Cevidanes LH, Bailey LJ, Tucker GR (2005). Superimposition of 3D cone-beam CT models of orthognathic surgery patients. Dentomaxillofac Radiol.

[17] An S, Lee JY, Chung CJ, Kim KH (2017). Comparison of different midsagittal plane configurations for evaluating craniofacial asymmetry by expert preference. Am J Orthod Dentofac Orthop.

[18] Cantarella D, Dominguez-Mompell R, Moschik C (2018). Midfacial changes in the coronal plane induced by microimplant-supported skeletal expander, studied with cone-beam computed tomography images. Am J Orthod Dentofac Orthop.

[19] Abedini S, Elkenawy I, Kim E, Moon W (2018). Three-dimensional soft tissue analysis of the face following micro-implant-supported maxillary skeletal expansion. Prog Orthod.

[20] Faul F, Erdfelder E, Lang AG, Buchner A (2007). G*Power 3: a flexible statistical power analysis program for the social, behavioral, and biomedical sciences. Behav Res Methods.

